# Predictive potential of distance-related spectral graphical descriptors for structure-property modeling of thermodynamic properties of polycyclic hydrocarbons with applications

**DOI:** 10.1038/s41598-024-72877-z

**Published:** 2024-09-28

**Authors:** Sakander Hayat, Seham J. F. Alanazi, Muhammad Imran, Muhammad Azeem

**Affiliations:** 1 Mathematical Sciences, Faculty of Science, Universiti Brunei Darussalam, Jln Tungku Link, Gadong, BE1410 Brunei Darussalam; 2 Department of Chemistry, College of Science (CS), King Saud University, 11451 Riyadh, Saudi Arabia; 3 Department of Mathematical Sciences, College of Science, United Arab Emirate University, Al Ain, 15551 UAE; 4 Department of Solids and Structures, School of Engineering, The University of Manchester, Oxford Road, Manchester, M13 9PL UK; 5 Department of Mathematics, Riphah International University, Lahore, Pakistan

**Keywords:** Mathematical chemistry, Distance-related spectral descriptors, Entropy, Heat capacity, Polycyclic hydrocarbons, Structure-property models, Carbon nanostructures, Cheminformatics, Theoretical chemistry, Applied mathematics

## Abstract

A distance-related spectral descriptor is a graphical index with defining structure built on eigenvalues of chemical matrices relying on distances in graphs. This paper explores the predictive ability of both existing and new distance-related spectral descriptors for estimating thermodynamic characteristics of polycyclic hydrocarbons (PHs). As a standard choice, the entropy and heat capacity are selected to represent thermodynamic properties. Furthermore, 30 initial members of PHs are considered as test molecules for this study. Three new molecular matrices have been proposed and our research demonstrates that distance-spectral graphical indices built by these novel matrices surpass in efficiency relative to famous distance-spectral indices. First, a novel computational method is put forwarded to evaluate distance-spectral indices of molecular graphs. The proposed methodology is utilized to compute both pre-existing and novel distance-related spectral descriptors, with an aim to assess their predictive efficacy using experimental data pertaining to two selected thermodynamic properties. Subsequently, we identify the five most promising distance-related spectral descriptors, comprising the degree-distance and Harary energies, the recently introduced second geometric-arithmetic energy along with its associated Estrada invariant, and 2$\text {nd}$ atom-bond connectivity (ABC) Estrada index. Notably, the 2$\text {nd}$ ABC Estrada index and Harary energy demonstrate correlation coefficients exceeding 0.95, while certain conventional spectral indices including the distance energy as well as its associated Estrada index, display comparatively lower performance levels. Moreover, we illustrate the practical implications of our findings on specific classes of one-hexagonal nanocones and carbon polyhex nanotubes. These outcomes hold potential for enhancing the theoretical determination of certain thermodynamic attributes of these nanostructures, offering improved accuracy and minimal margin of error.

## Introduction

Quantitative Structure-Property Relationship methodologies, also known as QSPR modeling, offer an effective means of establishing correlations between the physicochemical attributes and biological activities of chemical compounds. Graphical topological indices/descriptors/invariants play a pivotal role in constructing robust regression models with high predictive capability. Harold Wiener, whose ground-breaking research dates back to 1947, introduced^1^ structure-based topological descriptors, initially proposing the concept of the path number as the sum of geodesics between pairs of vertices within that graph. Subsequently designated as the Wiener index, this structurally invariant path number was observed to exhibit correlation with the boiling points of alkanes. The Wiener index represents the first example of a topologically-invariant graphical descriptor.

Transforming a chemical graph into a numerical representation is achieved through structurally-invariant measures termed as topological indices or descriptors. These indices capture structural information of a molecular graph, which depicts a hydrogen-deficient molecular configuration wherein edges denote chemical bonds and vertices represent atoms within organic chemical compounds. This area of investigation is recognized as chemical graph theory. Subsequently, physicochemical attributes including critical temperature, normal boiling point, critical volume, enthalpy of vaporization, critical pressure, standard heat of formation, among others, are then correlated utilizing these graphical indices. This correlation facilitates the development of regression models with robust predictive capabilities.

Prominent categories of graphical indices encompass distance-related indices^2^, degree/valency-related graphical invariants^3^, eigenvalues/spectrum-related graphical descriptors^4^, and counting-related structural polynomials and invariants^5^. Eigenvalues-related structure descriptors deliver structure-property models of notable efficacy across a spectrum of physical-chemical attributes, such as $\pi$-electronic energy ($E_{\pi }$), predicated upon the eigenvalues of specific chemical matrices. To date, a plethora of molecular descriptors^6,7^ have been posited. Nonetheless, the proliferation of molecular descriptors persists unabated due to the lack of stringent criteria to impede or circumscribe their proliferation. A considerable proportion of these structural invariants are graphical indices (graph-based topological descriptors). Consequently, lacking a robust criterion to arrest or at least mitigate the proliferation, the number of molecular descriptors far exceeds necessity.

To retard the proliferation of degree-related graphical invariants, Gutman et al.^8^ undertook a comparative assessment concerning octanes’ isomers. Hayat et al.^9^ took place a similar comparative testing for lower PHs with the aim of impeding the expansion of distance-related topological descriptors. The predictive capability of degree-based invariants, distance-related invariants, and the Estrada index in correlating $\pi$-electronic energy has been investigated in Refs.^10,11^ and Refs.^12,13^ respectively.

To advance the objective of diminishing the array of suggested graph-theoretic molecular structure descriptors, it is imperative to undertake comparative assessments. Additionally, we aim to highlight descriptors exhibiting superior correlation capabilities, warranting their incorporation into quantitative structure-property relationship models. Such descriptors of optimal performance demand heightened scrutiny from researchers within the domains of chemical graph theory and theoretical chemistry. The present study is centered on examining distance-related spectral descriptors.

The paper is organized as follows: Sect. "Mathematical preliminaries" delivers all the distance spectral descriptors. Our computational method is explained in Sect. "A computer-dependent computational technique". Section "Predictive ability of distance-related spectral descriptors" conducts the comparative testing to outline the best five spectral descriptors in predicting thermodynamic properties. Application of the five best spectral descriptors are presented for families of hexagonal polyhex nanotubes (resp. hexagonal nanocones) in Sect. "Applicability to carbon nanostructures: carbon nanotubes" (resp. Applicability to carbon nanostructures: one-hexagonal nanocones). We conclude the paper and present suitable regression models for the best five distance-spectral descriptors in Sect. Discussion and concluding remarks.

## Mathematical preliminaries

From a mathematical perspective, a graph denoted as $\Pi$ can be formally characterized as an ordered pair $\Pi =(V,E)$, wherein $V=V(\Pi )$ signifies a collection of points termed vertices, while $E(\Pi )\subseteq {V\atopwithdelims ()2}$ delineates the associations between points’ pairs termed as edges, Specifically, a chemical or molecular graph portrays a hydrogen-suppressed molecular structure derived from a compound. In this context, vertices (and edges) within a molecular graphical structure respective to atoms (and bonds) within the fundamental chemical compound. Two vertices are designated as adjacent if there exists an edge linking them. The count of adjacent vertices to a specified vertex $y\in V(\Pi )$ is termed the degree or valency of *y* and is represented as $d_y$. In the representation of an organic compound through a chemical graph, the degree of a vertex, corresponding to a carbon atom, is inherently limited to a maximum of four, corresponding to a carbon atom’s valency. For further elucidation on undefined notions pertaining to the theory of molecular graphs, we recommend consulting^14,15^.

A molecular graphical index is a transformations sending a molecular graph to a real number such that it finds significant chemical applicability^16^. Valency or degree-dependent graphical descriptors are distinguished by their structural characteristics, which are determined by the valencies of vertices. In contrast, distance-related topological descriptors are defined by the geodesics among vertices within a graph. Instances of degree-dependent graphical descriptors encompass the sum-connectivity, the atom-bond connectivity (ABC), the Randić and the two classical Zagreb invariants. Whereas, the Szeged, Balaban and Wiener are some famous instances of distance-dependent graphical descriptors. Eigenvalues-related structure invariants involve the establishment of structures based on the eigenvalues of matrices which are both graphical and molecular. Included in this category are spectral invariants like the adjacency energy and Estrada indices, the distance energy, and Laplacian and signless Laplacian energies, along with the corresponding Estrada indices, among numerous others. Hosamani et al.^17^ offer a comprehensive review of various findings concerning energies derived from certain graphical matrices. For further exploration of graph energy, readers are directed to the book by Li et al.^18^. Additionally, for a comprehensive treatment of the spectral radius of various graphical matrices, reference is made to the book by Soltani et al.^19^. Computational findings pertaining to distance-valency-related graphical indices are recorded in Refs.^9,20–22^. For structure-property modeling of physicochemical properties of special drugs and medicinal compounds, we refer to Refs.^23–28^.

Following this, we present several established spectral topological descriptors associated with distances in graphs, derived from the spectra of chemical matrices.

### The distance matrix

This matrix is purely distance-based and finds its roots in the work of Arthur Cayley^29^, where it was employed within a geometric framework. Its formal exploration within graph theory commenced in the 20th century, as elucidated in Ref.^30^. Let

$$\begin{aligned} \delta _\mathscr {D}^-=|\{\delta :\delta ~\mathrm {is~an~eigenvalue~of~}\mathscr {D},~\delta <0\}|. \end{aligned}$$

Graham et al.^31^ generated a correlation between $\delta _\mathscr {D}^-$ and the resolution of the addressing predicament in data communication systems.

The distance matrix $\mathscr {D}$, denoted as $\mathscr {D}_{\Pi }$, is associated with an $\upsilon$-vertex graph $\Pi$ and is represented as an $\upsilon \times \upsilon$ symmetric matrix. It is defined by,

$$\begin{aligned} (\mathscr {D}_{\Pi })_{w,z}=\left\{ \begin{array}{ll} d_{w,z}, & w\ne z; \\ 0, & w=z, \end{array} \right. \end{aligned}$$

here, the distance/geodesic between vertices *w* and *z* in $\Pi$ is symbolized as $d_{w,z}$. Let $\delta ^\mathscr {D}_1 \ge \ldots \ge \delta ^\mathscr {D}_n$ represent the eigenvalues of $\mathscr {D}$, referred to as the $\mathscr {D}$-eigenvalues of $\Pi$.

By the Frobenius theorem, we obtain $\delta ^\mathscr {D}_1>0$. The distance spectral radius of $\Pi$ is mathematically written as:

1 $$\begin{aligned} \rho _\mathscr {D}(\Pi )=\rho _\mathscr {D}:=\delta ^\mathscr {D}_1 \end{aligned}$$

Indulal^32^ undertook an investigation concerning $\rho _\mathscr {D}$ of graphs. Zhou et al.^33^ established extreme findings pertaining to $\rho _\mathscr {D}$ and delved into its chemical implications.

Furthermore, another topological descriptor based on spectra that corresponds to the distance matrix of graphs is the distance energy. This metric has defining structure as the summation of the absolute terms of the $\mathscr {D}$-eigenvalues of graphs.

2 $$\begin{aligned} En_\mathscr {D}=En_\mathscr {D}(\Pi ):=\sum \limits _{i=1}^{\upsilon }|\delta ^\mathscr {D}_i| \end{aligned}$$

A comprehensive study was conducted by Indulal et al.^34^ on $En_\mathscr {D}$ of graphs, deriving certain extremal results for them. Similarly, in their respective inquiries, Indulal^32^ and Zhou and Ilić^33^ have contributed by delineating precise upper and lower boundaries concerning distance energy, while identifying the associated extremal graphs. Additionally, Bozkurt et al.^35^ delved into the examination graphs which are distance equienergetic.

The $\mathscr {D}$-Estrada index is characterized by the distance metric, which is articulated as follows:

3 $$\begin{aligned} Es_\mathscr {D}=Es_\mathscr {D}(\Pi ):=\sum \limits _{i=1}^{\upsilon }\exp (\delta ^{\mathscr {D}}_i) \end{aligned}$$

The notion of $Es_\mathscr {D}$ was further studied by Güngör et al.^36^, providing bounds and establishing its correlation with $En_\mathscr {D}$ of graphs. Lately, Shang^37^ proposed a technique to approximate $Es_\mathscr {D}$ for graphs and applied it to the carbon allotrope, buckminsterfullerene $C_{60}$.

For a comprehensive understanding of the distance eigenvalues of graphs, we suggest consulting the exhaustive survey conducted by Aouchiche and Hansen^38^ on the distance spectrum of graphs.

#### The distance Laplacian

In the context of an $\upsilon$-vertex graph denoted as $\Pi$, we define the transmission of a vertex $w \in \Pi$ as the aggregate of distances between the designated vertex *w* and all remaining vertices within $\Pi$.

$$\begin{aligned} Tr(w):=\sum \limits _{z\in Z(\Pi )}d_{\Pi }(w,z). \end{aligned}$$

The mean transmission, denoted as $t(\Pi )$, for a graph $\Pi$ is articulated as follows:

$$\begin{aligned} t(\Pi ):=\frac{1}{\upsilon }\sum \limits _{z\in Z(\Pi )}Tr(z). \end{aligned}$$

The aggregate transmission of a graph denoted as $\Pi$ is formally delineated as the cumulative sum of distances between every unordered pair of vertices within $\Pi$. It is evident that

$$\begin{aligned} \sigma (\Pi ):=\frac{1}{2}\sum \limits _{z\in Z(\Pi )}\textbf{Tr}(z). \end{aligned}$$

The $\Pi$ transmission vector, represented as an $\upsilon \times 1$ vector $\textbf{Tr}$, is defined as $\textbf{Tr}_{w}:=Tr(w)$ for every $w\in Z(\Pi )$. Let $\textrm{Diag}(\textbf{Tr})$ denote the diagonal matrix associated with $\textbf{Tr}$, such that $\textrm{Diag}(\textbf{Tr}){yy}:=\textbf{Tr}(w)$. The $\Pi$ distance Laplacian matrix, denoted $\mathscr {L}$, is a symmetric $\upsilon \times \upsilon$ matrix defined as

$$\begin{aligned} \mathscr {L}=\textrm{Diag}(\textbf{Tr})-\mathscr {D}, \end{aligned}$$

where $\mathscr {D}$ represents the distance matrix of $\Pi$. This matrix was initially proposed by Aouchiche and Hansen^39^ and serves as the Laplacian-analogue of the classical adjacency matrix.

Let us posit that $\delta ^\mathscr {L}_1\ge \delta ^\mathscr {L}_2\ge \ldots \ge \delta ^\mathscr {L}_\upsilon =0$ represents the eigenvalues of the distance Laplacian matrix $\mathscr {L}$ associated with a graph $\Pi$. These eigenvalues are referred to as the $\mathscr {L}$-eigenvalues.

Consequently, the greatest $\mathscr {L}$-eigenvalue is termed as the $\mathscr {L}$-spectral radius.

4 $$\begin{aligned} \rho _\mathscr {L}=\rho _\mathscr {L}(\Pi ):=\delta ^\mathscr {L}_1 \end{aligned}$$

Aouchiche and Hansen^39^ have established several ground-breaking findings regarding the maximum $\rho _\mathscr {L}$ of the $\mathscr {L}$-matrix of graphs.

Defined by applying the summation on the absolute terms of $\delta ^\mathscr {L}_i~(1\le i\le n)$, the distance-Laplacian energy or $\mathscr {L}$-energy $E_\mathscr {L}$ was delivered by Das and colleagues^40^. Between $E_\mathscr {L}$ and $En_{\mathscr {D}}$, they also derived possible connections. Thus, the parameter $E_\mathscr {L}$ is mathematically defined as:

5 $$\begin{aligned} E_\mathscr {L}=E_\mathscr {L}(\Pi ):=\sum \limits _{i=1}^{\upsilon }|\delta ^\mathscr {L}_i-t(\Pi )| \end{aligned}$$

In 2015, Shang^41^ put forwarded the notion of the $\mathscr {L}$-Estrada index $Es_{\mathscr {L}}$ of graphs. Subsequently, $Es_{\mathscr {L}}$ is delineated in the ensuing expression.

6 $$\begin{aligned} Es_\mathscr {L}=Es_\mathscr {L}(\Pi ):=\sum \limits _{i=1}^{\upsilon }e^{(\delta ^\mathscr {L}_i-\sigma (\Pi ))} \end{aligned}$$

Specifically, Shang^41^ investigated evolving graphs in relation to their $Es_{\mathscr {L}}$.

#### The distance signless Laplacian

Introduced as the distance-analogue of the classical signless Laplacian of a graph, Aouchiche et al.^39^ proposed the distance signless Laplacian $\mathscr {Q}$.

The $\mathscr {Q}$ matrix of $\Pi$ pertaining to a graph $\Pi$ with $\upsilon$ vertices, is formally has the following square symmetric structure.

$$\begin{aligned} \mathscr {Q}=\textrm{Diag}(\textbf{Tr})+\mathscr {D}. \end{aligned}$$

In this context, $\textbf{Tr}$ represents the transmission vector, while $\mathscr {D}$ denotes the distance matrix pertaining to the set $\Pi$.

Let us posit that the $\mathscr {Q}$-eigenvalues be symbolized as $\delta ^\mathscr {Q}_1\ge \delta ^\mathscr {Q}_2\ge \ldots \ge \delta ^\mathscr {Q}_\upsilon$, henceforth referred to as the $\mathscr {Q}$-eigenvalues, pertaining to a graph $\Pi$. The greatest $\mathscr {L}$-eigenvalue is termed as the $\mathscr {L}$-spectral radius

7 $$\begin{aligned} \rho _\mathscr {Q}=\rho _\mathscr {Q}(\Pi ):=\delta ^\mathscr {Q}_1 \end{aligned}$$

Aouchiche and Hansen^42^ presented several significant findings regarding extremal properties pertaining to the parameter $\rho _{\mathscr {Q}}$ of graphs. Subsequently, Medina et al.^43^ derived precise upper extreme values for $\rho _{\mathscr {Q}}$, and further identified the specific graphs that attain these upper extremes.

Proposed by Das et al.^40^, the $\mathscr {Q}$-energy $En_{\mathscr {Q}}$ is obtained by applying the summation on the absolute terms of $\delta ^\mathscr {Q}_i~(1\le i\le n)$.

8 $$\begin{aligned} En_\mathscr {Q}=En_\mathscr {Q}(\Pi ):=\sum \limits _{i=1}^{\upsilon }|\delta ^\mathscr {Q}_i-t(\Pi )| \end{aligned}$$

Medina et al.^43^, Alhevaz et al.^44^, and Alhevaz et al.^45^ achieved specific extremal findings concerning the $En_{\mathscr {Q}}$ of graphs.

Alhevaz et al.^46^ have recently proposed the concept of the $\mathscr {Q}$-Estrada index for graphs, thereby combining the spectral frameworks of both the $Es_{\mathscr {D}}$ and $Es_{\mathscr {Q}}$.

9 $$\begin{aligned} Es_\mathscr {Q}=Es_\mathscr {Q}(\Pi ):=\sum \limits _{i=1}^{\upsilon }e^{(\delta ^\mathscr {Q}_i-\sigma (\Pi ))} \end{aligned}$$

Alhevaz and coauthors^46^ joined the theories of $Es_{\mathscr {D}}$ and $Es_{\mathscr {Q}}$. Alhevaz et al.^45^ established precise extreme values of $Es_{\mathscr {Q}}$, and identified the graphs achieving the extreme values.

#### The Harary matrix

The Harary matrix, as presented by Güngör and Çevic^47^, has been a recent addition to the academic discourse. In the context of a graph denoted by $\Pi$, comprising $\upsilon$ vertices, the Harary matrix, denoted as $H_{\Pi }$, is established as an $\upsilon \times \upsilon$ symmetric matrix, defined as follows:

$$\begin{aligned} (H_{\Pi })_{w,z}=\left\{ \begin{array}{ll} \frac{1}{d_{\Pi }(w,z)}, & w\ne z; \\ 0, & w=z. \end{array} \right. \end{aligned}$$

Let $\delta ^{H}_1\ge \ldots \ge \delta ^{H}_\upsilon$ denote the eigenvalues of a graph *H*, henceforth referred to as *H*-eigenvalues, within the context of a graph $\Pi$. The *H*-spectral radius is delineated as the maximum among its Harary eigenvalues.

10 $$\begin{aligned} \rho _{H}=\rho _{H}(\Pi ):=\delta ^{H}_1 \end{aligned}$$

The energy and Estrada index for the *H*-matrix were initially put forwarded and examined by Güngör and Çevic^47^. In the context of a graph $\Pi$, these metrics are delineated as follows:

11 $$\begin{aligned} & En_{H}=En_{H}(\Pi ):=\sum \limits _{i=1}^{\upsilon }|\delta ^{H}_i| \end{aligned}$$

12 $$\begin{aligned} & Es_{H}=Es_{H}(\Pi ):=\sum \limits _{i=1}^{\upsilon }e^{\delta ^{H}_i} \end{aligned}$$

Cui and Liu^48^ conducted an examination into distinct characteristics concerning the Harary eigenvalues of graphs. Furthermore, they formulated both upper and lower limits pertaining to $Es_{H}$ and $En_{H}$ of graphs. Additionally, Jahanbani^49^ introduced novel constraints on $Es_{H}$ and $En_{H}$ of graphs.

#### The Szeged matrix

Prior to delineating the Szeged matrix, it is imperative to establish several foundational definitions. In association with an edge $e=wz\in E(\Pi )$ within a graph $\Pi$, we introduce the ensuing parameters:

$$\begin{aligned} q_{w,e}:= & \mid \{x\in V(\Pi )\mid d_{\Pi }(x,w)<d_{\Pi }(w,z)\}\mid ,\\ q_{z,e}:= & \mid \{x\in V(\Pi )\mid d_{\Pi }(x,w)>d_{\Pi }(w,z)\}\mid ,\\ q_{0,e}:= & \mid \{x\in V(\Pi )\mid d_{\Pi }(x,w)=d_{\Pi }(w,z)\}\mid . \end{aligned}$$

Based on the quantities $q_{w,e}$ and $q_{z,e}$, Diudea et al.^50^ introduced the Szeged matrix of graphs. For an $\upsilon$-vertex graph $\Pi$, the Szeged matrix $Sz=Sz_{\Pi }$ is an $\upsilon \times \upsilon$ symmetric matrix defined as: For an $\upsilon$-vertex graph denoted as $\Pi$, the Szeged matrix *Sz*, denoted as $Sz_{\Pi }$, is defined as an $\upsilon \times \upsilon$ symmetric matrix as follows:

$$\begin{aligned} (\mathscr{S}\mathscr{Z}_{\Pi })_{w,z}=\left\{ \begin{array}{ll} q_{w,e}q_{z,e}, & wz\in E(\Pi ); \\ 0, & \hbox {Otherwise.} \end{array} \right. \end{aligned}$$

Let $\delta ^\mathscr{S}\mathscr{Z}_1\ge \ldots \ge \delta ^\mathscr{S}\mathscr{Z}_\upsilon$ denote the eigenvalues of the Szeged matrix, denoted as *Sz*, associated with a graph $\Pi$. These eigenvalues are commonly referred to as the Szeged eigenvalues or simply *Sz*-eigenvalues. Fath-Tabar et al.^51^ explored the properties of Szeged eigenvalues and their associated spectral descriptors within the context of graphs. They presented several extremal results pertaining to the Szeged eigenvalues of graphs. The Szeged spectral radius is defined as the maximum eigenvalue of the Szeged matrix associated with a graph.

13 $$\begin{aligned} \rho _\mathscr{S}\mathscr{Z}=\rho _\mathscr{S}\mathscr{Z}(\Pi ):=\delta ^\mathscr{S}\mathscr{Z}_1 \end{aligned}$$

Alternatively, the Szeged energy of a graph is calculated by summing $|\delta ^\mathscr {Q}_i~(1\le i\le n)|$.

14 $$\begin{aligned} En_\mathscr{S}\mathscr{Z}=En_\mathscr{S}\mathscr{Z}(\Pi ):=\sum \limits _{i=1}^{\upsilon }|\delta ^\mathscr{S}\mathscr{Z}_i| \end{aligned}$$

On a similar note, the Szeged Estrada index has the following defining structure:

15 $$\begin{aligned} Es_\mathscr{S}\mathscr{Z}=Es_\mathscr{S}\mathscr{Z}(\Pi ):=\sum \limits _{i=1}^{\upsilon }e^{\delta ^\mathscr{S}\mathscr{Z}_i} \end{aligned}$$

Although the Szeged spectrum and it associated distance-spectral invariants are not well-studied, finding their potential applicability in structure-property modeling of thermodynamic characteristics is worth investigating.

#### The Padmakar-Ivan (PI) matrix

The PI matrix and its associated distance-spectral invariants were proposed and studied by Najdafi-Arani^52^ in 2011. The PI matrix, denoted as $\mathscr{P}\mathscr{I}=PI_{\Pi }$ for a graph $\Pi$ with $\upsilon$ vertices, is defined as a symmetric $\upsilon \times \upsilon$ matrix.

$$\begin{aligned} (PI_{\Pi })_{w,z}=\left\{ \begin{array}{ll} q_{w,e}+q_{z,e}, & {wz\in E(\Pi );} \\ 0, & \hbox {Otherwise.} \end{array} \right. \end{aligned}$$

The eigenvalues of the matrix *PI* are denoted by $\delta ^{PI}_1\ge \ldots \ge \delta ^{PI}_\upsilon$, referred to as the PI-eigenvalues of the graph $\Pi$. The PI spectral radius is defined as the maximum PI-eigenvalue for a given graph.

16 $$\begin{aligned} \rho _{PI}=\rho _{PI}(\Pi ):=\delta ^{PI}_1 \end{aligned}$$

The PI energy of a graph is the total sum of the absolute values of its PI-eigenvalues.

17 $$\begin{aligned} En_{PI}=En_{PI}(\Pi ):=\sum \limits _{i=1}^{\upsilon }|\delta ^{PI}_i| \end{aligned}$$

Similarly, the PI Estrada index is defined using the following expression.

18 $$\begin{aligned} Es_{PI}=Es_{PI}(\Pi ):=\sum \limits _{i=1}^{\upsilon }e^{\delta ^{PI}_i} \end{aligned}$$

Najdafi-Arani demonstrated some precise limits on the PI energy of graphs in their work^52^. They also identified the corresponding extremal graphs in the same study.

#### The degree-distance matrix

Dobrynin et al.^53^ introduced the degree-distance matrix concerning the degree-distance index of graphs. The degree-distance matrix $DD=DD_{\Pi }$ of an $\upsilon$-vertex graph $\Pi$ is an $\upsilon \times \upsilon$ symmetric matrix defined as follows:

$$\begin{aligned} (\mathscr{D}\mathscr{D}_{\Pi })_{w,z}=\left\{ \begin{array}{ll} \frac{d_w+d_z}{d_{\Pi }(w,z)}, & w\ne z; \\ 0, & w=z. \end{array} \right. \end{aligned}$$

The eigenvalues of the matrix *DD* are denoted by $\delta ^{DD}_1\ge \ldots \ge \delta ^{DD}_\upsilon$, referred to as the *DD*-eigenvalues for a graph $\Pi$. The degree-distance spectral radius, energy, and Estrada index are defined as follows:

19 $$\begin{aligned} & \rho _{DD}=\rho _{DD}(\Pi ):=\delta ^{DD}_1 \end{aligned}$$

20 $$\begin{aligned} & En_{DD}=En_{DD}(\Pi ):=\sum \limits _{i=1}^{\upsilon }|\delta ^{DD}_i| \end{aligned}$$

21 $$\begin{aligned} & Es_{DD}=Es_{DD}(\Pi ):=\sum \limits _{i=1}^{\upsilon }e^{\delta ^{DD}_i} \end{aligned}$$

Kanna et al. presented some precise lower and upper bounds on the degree-distance energy of graphs in their work^54^.

#### The Schultz matrix

Let $A_{\Pi }$ denote the adjacency matrix of $\Pi$, and $D_{\Pi }$ denote the distance matrix of $\Pi$. Schultz^55^ defined the Schultz matrix as follows:

22 $$\begin{aligned} \mathscr {S}_{\Pi }=A_{\Pi }+D_{\Pi }. \end{aligned}$$

The Schultz matrix, as introduced by Schultz^55^, has shown promise in predicting the normal boiling points of alkanes and other organic compounds.

The eigenvalues of *S* are denoted as $\delta ^\mathscr {S}_1\ge \ldots \ge \delta ^\mathscr {S}_\upsilon$, referred to as *S*-eigenvalues or simply Schultz eigenvalues, for a graph $\Pi$. Leveraging the spectrum-based indices for matrices defined earlier, we have explored the chemical relevance of the Schultz spectral radius, energy, and Estrada index. The Schultz spectral radius is defined as the largest Schultz eigenvalue of a graph.

23 $$\begin{aligned} \rho _\mathscr {S}=\rho _\mathscr {S}(\Pi ):=\delta ^\mathscr {S}_1 \end{aligned}$$

The Schultz energy and Schultz Estrada index are defined by the following expressions.

24 $$\begin{aligned} & En_\mathscr {S}=En_\mathscr {S}(\Pi ):=\sum \limits _{i=1}^{\upsilon }|\delta ^\mathscr {S}_i| \end{aligned}$$

25 $$\begin{aligned} & Es_\mathscr {S}=Es_\mathscr {S}(\Pi ):=\sum \limits _{i=1}^{\upsilon }e^{\delta ^\mathscr {S}_i} \end{aligned}$$

### Some new chemical matrices

In this subsection, we add some recently-introduced novel chemical matrices and evaluate their suitability in QSPR studies. They were introduced by Hayat et al.^56^. It is noteworthy that these matrices align with established distance-related topological descriptors of graphs.

#### The second atom-bond connectivity $(ABC^2)$ matrix

The definition of the general atom-bond connectivity index $ABC_k$, where *k* is a positive integer, for a graph $\Pi$ is as follows:

$$\begin{aligned} ABC_k(\Pi )=\sum \limits _{yz\in E(\Pi )}\sqrt{\frac{q_w+q_z-2}{q_wq_z}}. \end{aligned}$$

When $k=1$, the pair ($q_w,q_z$) equals ($d_w,d_z$), where $d_w$ represents the degree or valency of vertex *w* in graph $\Pi$. The initial *ABC* index was introduced by Estrada et al.^57^. Graovac et al.^58^ examined ($q_w,q_z$)=($q_{w,e},q_{z,e}$) and labeled it as the second iteration of the ABC index.

Acknowledging the reliance of numerous chemical matrices on corresponding topological descriptors, we introduce a chemical matrix grounded in the second atom-bond connectivity index. Hence, the second atom-bond connectivity matrix $ABC^2=ABC^2_{\Pi }$ of an $\upsilon$-vertex graph $\Pi$ is an $\upsilon \times \upsilon$ symmetric matrix defined as:

$$\begin{aligned} (\mathscr {A}\mathscr {B}\mathscr {C}^2_{\Pi })_{w,z}=\left\{ \begin{array}{ll} \sqrt{\frac{q_{w,e}+q_{z,e}-2}{q_{w,e}q_{z,e}}}, & {wz\in E(\Pi );} \\ 0, & \hbox {Otherwise.} \end{array} \right. \end{aligned}$$

The eigenvalues of $ABC^2$ are denoted by $\delta ^{ABC^2}_1\ge \ldots \ge \delta ^{ABC^2}_\upsilon$, referred to as $ABC^2$-eigenvalues, for a graph $\Pi$. The second ABC spectral radius is defined as the largest $ABC^2$-eigenvalue of a graph.

26 $$\begin{aligned} \rho _{ABC^2}=\rho _{ABC^2}(\Pi ):=\delta ^{ABC^2}_1 \end{aligned}$$

On the other hand, the second ABC energy and Estrada index are defined by the following equations.

27 $$\begin{aligned} & En_{ABC^2}=En_{ABC^2}(\Pi ):=\sum \limits _{i=1}^{\upsilon }|\delta ^{ABC^2}_i| \end{aligned}$$

28 $$\begin{aligned} & Es_{ABC^2}=Es_{ABC^2}(\Pi ):=\sum \limits _{i=1}^{\upsilon }e^{\delta ^{ABC^2}_i} \end{aligned}$$

In the sections to follow, we investigate the potential chemical utility of the second ABC matrix alongside associated spectrum-based topological descriptors.

#### The second geometric-arithmetic $(\mathscr{G}\mathscr{A}^2)$ matrix

The definition of the general geometric-arithmetic (GA) index $GA_k$, where *k* is a positive integer, for a graph $\Pi$ is as follows:

$$\begin{aligned} GA_k(\Pi )=\sum \limits _{wz\in E(\Pi )}\frac{2\sqrt{\ell _w\ell _z}}{\ell _w+\ell _z}. \end{aligned}$$

When ($\ell _w,\ell _z$)=($d_w,d_z$), *k* equals 1. The $GA_1$ index was introduced by Vukičevi’c et al.^59^. Fath-Tabar et al.^60^ introduced the second version of the GA index by considering ($\ell _w,\ell _z$)=($q_w,q_z$).

Derived from the second geometric-arithmetic index of an $\upsilon$-vertex graph $\Pi$, the second geometric-arithmetic matrix $GA^2=GA^2_{\Pi }$ is a symmetric $n\times n$ matrix defined as

$$\begin{aligned} (GA^2_{\Pi })_{w,z}=\left\{ \begin{array}{ll} \frac{2\sqrt{q_{w,e}q_{z,e}}}{q_{w,e}+q_{z,e}}, & wz\in E(\Pi ); \\ 0, & \hbox {Otherwise.} \end{array} \right. \end{aligned}$$

The eigenvalues of $GA^2$ are denoted by $\delta ^{GA^2}_1\ge \ldots \ge \delta ^{GA^2}_\upsilon$, referred to as $GA^2$-eigenvalues or simply the second GA eigenvalues, for a graph $\Pi$. The second GA spectral radius is defined as the largest $GA^2$-eigenvalue of a graph.

29 $$\begin{aligned} \rho _{GA^2}=\rho _{GA^2}(\Pi ):=\delta ^{GA^2}_1 \end{aligned}$$

The equations defining the second GA energy and second GA Estrada index are as follows.

30 $$\begin{aligned} En_{GA^2}=En_{GA^2}(\Pi ):=\sum \limits _{i=1}^{\upsilon }|\delta ^{GA^2}_i| \end{aligned}$$

31 $$\begin{aligned} Es_{GA^2}=Es_{GA^2}(\Pi ):=\sum \limits _{i=1}^{\upsilon }e^{\delta ^{GA^2}_i} \end{aligned}$$

In the following sections, we will examine the potential chemical relevance of the second GA matrix and its associated spectrum-based topological descriptors.

#### The Gutman matrix

The Gutman index, proposed by Gutman in 1994^61^, serves as a multiplicative rendition of the degree-distance index. It is defined as:

$$\begin{aligned} \mathscr {G}\mathscr {U}\mathscr {T}(\Pi )=\sum \limits _{w,z\in V(\Pi ), w\ne z} d_wd_zd_{\Pi }(w,z). \end{aligned}$$

Drawing inspiration from the defining structure of the Gutman index, we introduce the Gutman matrix $Gut=Gut_{\Pi }$ for an $\upsilon$-vertex graph $\Pi$, which is an $\upsilon \times \upsilon$ symmetric matrix defined as:

$$\begin{aligned} (\mathscr {G}\mathscr {U}\mathscr {T}_{\Pi })_{w,z}=\left\{ \begin{array}{ll} \frac{d_wd_z}{d_{\Pi }(w,z)}, & w\ne z; \\ 0, & w=z. \end{array} \right. \end{aligned}$$

The eigenvalues of *Gut* are denoted by $\delta ^\mathscr {G}\mathscr {U}\mathscr {T}_1\ge \ldots \ge \delta ^\mathscr {G}\mathscr {U}\mathscr {T}_\upsilon$, referred to as *Gut*-eigenvalues or the Gutman eigenvalues, for a graph $\Pi$. The Gutman spectral radius, Gutman energy, and Gutman Estrada index are defined by the following expressions.

32 $$\begin{aligned} \rho _\mathscr {G}\mathscr {U}\mathscr {T}=\rho _\mathscr {G}\mathscr {U}\mathscr {T}(\Pi ):=\delta ^\mathscr {G}\mathscr {U}\mathscr {T}_1 \end{aligned}$$

33 $$\begin{aligned} En_\mathscr {G}\mathscr {U}\mathscr {T}=En_\mathscr {G}\mathscr {U}\mathscr {T}(\Pi ):=\sum \limits _{i=1}^{\upsilon }|\delta ^\mathscr {G}\mathscr {U}\mathscr {T}_i| \end{aligned}$$

34 $$\begin{aligned} Es_\mathscr {G}\mathscr {U}\mathscr {T}=Es_\mathscr {G}\mathscr {U}\mathscr {T}(\Pi ):=\sum \limits _{i=1}^{\upsilon }e^{\delta ^\mathscr {G}\mathscr {U}\mathscr {T}_i} \end{aligned}$$

Roshan et al.^62^ introduced a correlated matrix known as the Gutman covering matrix for graphs. They established precise upper and lower bounds on the Gutman covering energy of graphs.

## A computer-dependent computational technique

This section delves into computational methods, which are computer-based techniques utilizing available software packages to calculate various methodologies. The emphasis lies on determining specific graphical indices related to temperature within benzenoid systems. However, it’s worth noting that this method extends beyond benzenoid structures and can be applied to general chemical graphs that defy embedding in $\mathbb {R}^2$.

The TopoCluj software^63^ specializes in molecular topology analysis by calculating topological descriptors from chemical graphs using a variety of matrices. A diverse class of graphical matrices known as incidence, adjacency and the class of Cluj matrices could easily be handled by TopoCluj. For invariants’ investigation, TopolCluj offers computational analysis of parameters such as Group Vertex Mass (Mass), Atomic Charges and Sanderson Group Electronegativity (SGI). Integrated with functionality of MatLab^64^, it employs transformation from graphs to matrices and vice versa.

A computational platform tailored for tasks such as molecular modeling, HyperChem^65^ delivers a detailed functionality. Molecular dynamics simulations such as Langevin dynamic and Metropolis Monte Carlo simulations and thermal motion’s effect on chemical systems are facilitated by HyperChem. HyperChem integrates both quantum and molecular mechanical calculations employing diverse quantum techniques, having specific conditions for ab-initio computations. It further offers a user-friendly platform for chemical kinetic quantification and its modeling. Nevertheless, it may lack certain conventional equilibrium force field capabilities for capturing chemical reactions effectively. MatLab functions as a versatile quantitative software environment, employing matrices operations in diverse mathematical applicability. Beyond matrix analysis, MatLab finds utility in fields like video/image processing in computer graphics, and applicability in civil/mechanical engineering.

The process described in Sect. Mathematical preliminaries involves taking a molecular graph $\Pi$ as its input and generating spectral indices related to distances. Herein, the algorithmic steps have been articulated: 

1st step: Use HyperChem to create a visual representation of $\Pi$, which will produce a .hin file that matches the graph.

2nd step: Upload the .hin file for $\Pi$, then choose the adjacency matrix from the list of invariants, labeled as *A*. Afterward, proceed to create the .m file.

3rd step: Run the Matlab code by providing the generated .m file as input, which will generate all distance-based spectral invariants outlined in Sect. Mathematical preliminaries.

**Fig. 1:**
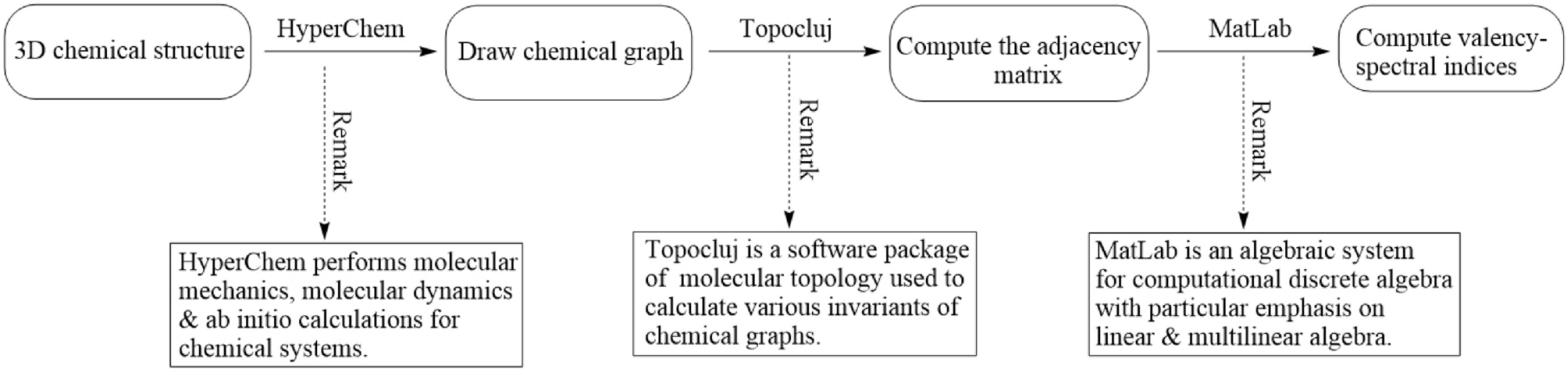
The figure presents a flowchart of our computer-based technique.

The provided figure, labeled as “Flowchart” (see Fig. 1), visually illustrates the structural flow of the proposed computing method.

## Predictive ability of distance-related spectral descriptors

We aim to explore the correlation power and assess the efficiency of distance-related graphical invariants from Sect. Mathematical preliminaries, in quantitative structure activity/property relationship studies. To achieve this, a comparative testing is carried out.

Entropy and heat capacity are the standard thermodynamic properties which are opted for any quality testing of graphical indices. These two thermodynamic attributes are then analysed in relation to a specific group of graphical invariants. The quality of the correlation value is directly proportional to the efficacy of a graphical index, has been chosen as a criteria to asses the quality. The experimental values of both entropy *Ent* and heat capacity $H_c$ has been retrieved from the standard NIST^66^ database.

**Fig. 2:**
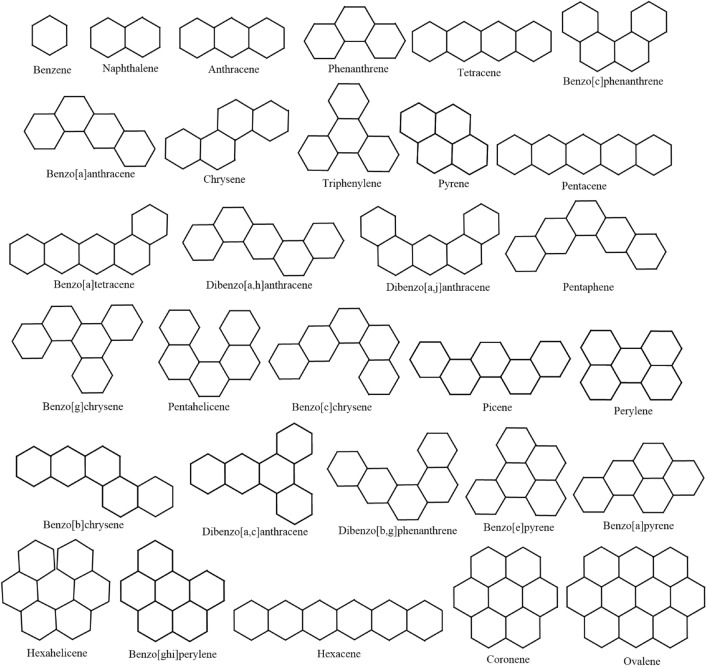
Graphical structures of the 30 test molecules.

Although conventional comparative tests often utilize isomeric alkanes as molecules, we have opted to employ lower polycyclic hydrocarbons (PHs). This choice stems from the fact that PHs encompass both cyclic and acyclic chemical structures, while isomeric alkanes predominantly represent acyclic molecular structures. This distinction is underscored by graph-theoretic reasoning, where trees, representing acyclic structures, are subsets of general graphs containing cycles. Additionally, the selection of PHs is motivated by the extension of spectrum-based topological descriptors, which transition from acyclic to cyclic structures. To ensure robust statistical deductions in our comparative analysis, it is imperative to have a sufficiently large dataset of molecules, coupled with publicly available experimental data. Hence, our selection of 30 initial PHs adheres to this criteria. Figure 2 illustrates the 30 PHs under consideration for the current study.

Regarding QSPR modeling of hydrocarbons, Fissa et al.^67^ studied QSPR-ANN models for certain critical properties of pure hydrocarbons. Flora et al.^68^ implemented machine learning in predicting ignition properties of certain hydrocarbons. Regarding mathematical methods in QSPR of hydrocarbons, Arockiaraj et al.^69^ implemented molecular hybrid geometric-harmonic-Zagreb degree based descriptors in structure-property studied of lower benzenoid hydrocarbons. Moreover, Raza et al.^70^ applied valency graphical descriptors in predicting physical properties of polycylic aromatic hydrocarbons.

The computational method outlined in Sect. "A computer-dependent computational technique" has been utilized to compute all distance-spectral invariants detailed in Sect. Mathematical preliminaries for the 30 lower PHs depicted by Fig. 2. Following this, we establish correlations between experimental values of *Ent* and $H_c$ and a graphical descriptor. Although, we aim to generate a curvilinear correlation, the attempt was unsuccessful in doing so for any of the distance-spectral graphical indices. Consequently, we assess the predictive potential of a graphical invariant by examining its correlated values with chosen thermodynamic characteristics. The correlation values with respect to heat capacity ($H_c$) and entropy (*Ent*) for lower PHs are presented in Table 1.

While the aim was to generate a curvilinear correlation, none could be developed for any of the distance-spectral invariants. Therefore, we assess the estimation capability of a graphical invariant by examining its Pearson correlated values with thermodynamic characteristics. Table 1 presents values illustrating these Pearson correlated values with heat capacity ($H_c$) and entropy (*Ent*) for lower PHs.

**Table 1:** Multiple correlation coefficients $\rho$($H_c,Ent$) of distance-spectral invariants with the $H_c$ and *Ent* for the 30 lower PHs.

Graphical index, $G_I$	$\rho (H_c,Ent;G_I)$
$\rho _D$, Eq. (1)	0.9795
$En_D$, Eq. (2)	0.9795
$Es_D$, Eq. (3)	0.7109
$\rho _{DL}$, Eq. (4)	0.9544
$En_{DL}$, Eq. (5)	0.9467
$\rho _DQ$, Eq. (7)	0.9778
$En_DQ$, Eq. (8)	0.9389
$Es_DQ$ Eq. (9)	0.6106
$\rho _S$, Eq. (23)	0.9797
$En_S$, Eq. (24)	0.9809
$Es_S$, Eq. (25)	0.7106
$\rho _{H}$, Eq. (10)	0.9647
$En_H$, Eq. (11)	0.9963
$Es_{H}$, Eq. (12)	0.8340
$\rho _{DD}$, Eq. (19)	0.9521
$En_{DD}$, Eq. (20)	0.9929
$Es_{DD}$, Eq. (21)	0.7081
$\rho _\mathscr {G}\mathscr {U}\mathscr {T}$, Eq. (32)	0.9395
$En_\mathscr {G}\mathscr {U}\mathscr {T}$, Eq. (33)	0.9876
$Es_\mathscr {G}\mathscr {U}\mathscr {T}$, Eq. (34)	0.7080
$\rho _\mathscr{S}\mathscr{Z}$, Eq. (13)	0.9143
$En_\mathscr{S}\mathscr{Z}$, Eq. (14)	0.9297
$\rho _{\Pi }$, Eq. (16)	0.9557
$En_{\Pi }$, Eq. (17)	0.9692
$Es_{\Pi }$, Eq. (18)	0.7080
$\rho _{ABC^2}$, Eq. (26)	0.6462
$En_{ABC^2}$, Eq. (27)	0.9703
$Es_{ABC^2}$, Eq. (28)	0.9941
$\rho _{GA^2}$, Eq. (29)	0.7447
$En_{GA^2}$, Eq. (30)	0.9924
$Es_{GA^2}$, Eq. (31)	0.9916

In Table 1, it’s imperative to note the strikingly close correlation coefficients between entropy and heat capacity, which are both tied to distance-related spectral descriptors. This suggests that despite their inherent differences, these thermodynamic properties are compatible. Consequently, they could serve as effective tools for evaluating the effectiveness of different topological or molecular descriptors in general.

## Applicability to carbon nanostructures: carbon nanotubes

Nanotechnology exploration commences as molecular structures shrink to the range of 1-100 nm. This field, boasting promising applicative potential medicine, computer science, and electronics, etc. fuels the creation of a plethora of innovative materials and devices. Carbon nanotubes (CNTs) are cylindrical nanostructures composed of carbon’s allotrope, stand out for their superior potential in practical applications when compared to other materials.

**Fig. 3:**
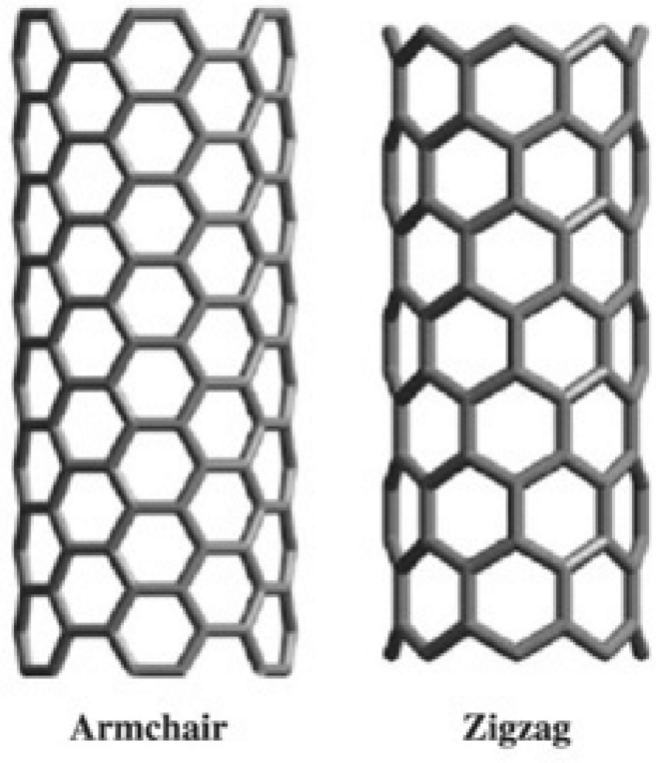
A visualization depicting polyhex nanotubes in both zigzag and armchair configurations from a three-dimensional perspective.

Carbon polyhex nanotubes feature a cylindrical surface defined by a hexagonal tessellation, exhibiting remarkable stability in natural environments. Their presence unlocks captivating thermal, electrical, and mechanical properties^71^. These distinctive attributes have propelled carbon polyhex nanotubes to become the most extensively researched nanostructures. Governed by chirality, these nanotubes manifest diverse shapes including zigzag, armchair, and chiral configurations. Refer to Fig. 3 for visual representations of the zigzag/armchair configurations of polyhex CNTs.

**Fig. 4:**
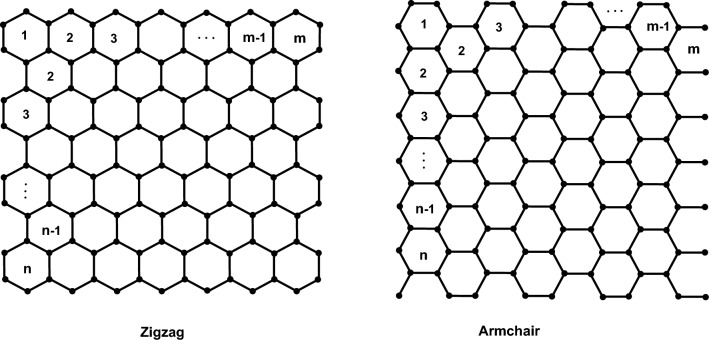
Polyhex nanotubes with zigzag and armchair configurations in (*m*, *n*) dimensions.

We aim to explore distance-spectral graphical invariants by generating 2D graphical structures for armchair/zigzag polyhex CNTs. The zigzag polyhex nanotube, designated as $TUZC_6[m,n]$, is characterized by two parameters, *n* and *m*, representing the count of hexagonal faces in each column and row, respectively. In a similar manner, a (*m*, *n*)-dimensional polyhex CNT is being symbolized as $TUAC_6[m,n]$, with *n* and *m* representing the count of hexagonal faces in each column and row, respectively. For simplicity, we use the notation $ZC_6[m,n]$ and $AC_6[m,n]$ to represent zigzag and armchair nanotubes with dimensions (*m*, *n*), respectively. Figure 4 showcases these polyhex-zigzag CNTs of dimensions (*m*, *n*).

This section utilizes the computing methodology outlined in Sect. A computer-dependent computational technique for computing the best 5 distance-spectral related invariants, as outlined in Table 5, for polyhex CNTs. Two infinite families of polyhex CNTs were considered, denoted as $ZC_6[m,n]$, with parameters $m=4$ and $m=5$. The same methodology can be extended to other cases. Specifically, Table 2 showcases the computed results for the polyhex CNT $ZC_6[4,n]$, where $2\le n\le 10$, excluding $ZC_6[4,1]$ due to its trivial nature.

**Table 2:** Identifying the best 5 distance-spectral invariant within the $ZC_6[4,n]$ structure, where *n* ranges from 2 to 15.

$ZC_6[4,n]$	$En_{H}$	$Es_{GA^2}$	$En_{GA^2}$	$En_{DD}$	$Es_{ABC^2}$
$ZC_6{[}4,2{]}$	33.9739	61.8001	33.0159	171.7260	31.1840
$ZC_6{[}4,3{]}$	49.5709	87.8085	47.0358	257.8670	43.8298
$ZC_6{[}4,4{]}$	55.4696	100.3755	53.8542	293.4041	48.1860
$ZC_6{[}4,5{]}$	75.1084	137.9933	72.8540	401.5943	61.2726
$ZC_6{[}4,6{]}$	85.3010	155.1719	81.9266	460.3674	68.9791
$ZC_6{[}4,7{]}$	100.7379	181.9450	96.5166	545.7397	80.7527
$ZC_6{[}4,8{]}$	110.8029	201.1642	106.5684	603.7799	88.2220
$ZC_6{[}4,9{]}$	126.3356	230.1936	121.6045	689.7301	99.6331
$ZC_6{[}4,10{]}$	136.5202	250.8521	132.2422	748.3675	106.8188
$ZC_6{[}4,11{]}$	152.0197	282.0789	147.9012	834.0704	118.1205
$ZC_6{[}4,12{]}$	162.1132	302.5011	158.2979	892.3617	125.3207
$ZC_6{[}4,13{]}$	177.6372	333.4137	173.9538	978.1854	136.5843
$ZC_6{[}4,14{]}$	187.7551	354.0322	184.5034	1036.5700	143.7324
$ZC_6{[}4,15{]}$	203.2384	385.2485	200.1778	1122.2477	154.9428

Additionally, we employ the cftool within MatLab to analyze the data presented in Table 2. The cftool serves as a resource for curve and surface fitting in MatLab. Our approximations were conducted using the R2013a version of MatLab. The subsequent results, accompanied by 95% confidence intervals for coefficients, were generated by our MatLab program:

$$\begin{aligned} En_{H}(ZC_6[4,n])= & 1408_{\pm 23700}\sin (0.009226_{\pm 0.15658}n + 0.0058_{\pm 0.09258}),\\ Es_{GA^2}(ZC_6[4,n]))= & 0.15_{\pm 0.32}n^2 + 22.1_{\pm 5.5}n + 17.32_{\pm 20.7},\\ En_{GA^2}(ZC_6[4,n])= & 0.05_{\pm 0.15}n^2 + 11.97_{\pm 2.68}n + 9.1_{\pm 10.1},\\ En_{DD}(ZC_6[4,n])= & 7898_{\pm 126780}\sin (0.009241_{\pm 0.14947}n +0.003321_{\pm 0.04871})\\ Es_{ABC^2}(ZC_6[4,n])= & -0.015_{\pm 0.124}n^2 + 9.68_{\pm 2.15}n + 12.5_{\pm 8.1} + 7.483 _{\pm 22.298}n +15.52{\pm 33.801}. \end{aligned}$$

These discoveries will aid in establishing a more precise correlation between the $\pi$-electron energies of $ZC_6[4,n]$.

Furthermore, we utilize our computational approach detailed in Sect. A computer-dependent computational technique to determine the best 5 distance-spectral graphical invariants as listed in Table 5 for the $ZC_6[5,n]$ nanotube. The resulting values for the polyhex-zigzag CNT $ZC_6[5,n]$ are tabulated in Table 3 for $2\le n\le 15$, excluding $ZC_6[5,1]$ due to its trivial nature.

**Table 3:** Optimal topological descriptors based on spectrum analysis for $ZC_6[5,n]$, where $2\le n\le 15$, are identified as the top five.

$ZC_6[5,n]$	$En_H$	$Es_{GA^2}$	$En_{GA^2}$	$En_{DD}$	$Es_{ABC^2}$
$ZC_6[5,2]$	42.4156	77.6273	41.0904	217.4252	37.7476
$ZC_6[5,3]$	60.9452	109.6806	58.1305	321.2256	52.1344
$ZC_6[5,4]$	74.0452	130.5868	69.7692	397.3709	62.2993
$ZC_6[5,5]$	92.5372	163.3578	87.0582	500.9904	76.2604
$ZC_6[5,6]$	105.5011	188.1582	99.8468	576.5361	85.7444
$ZC_6[5,7]$	123.9716	221.5539	117.2291	680.0174	99.4414
$ZC_6[5,8]$	137.1272	246.5397	130.2352	756.5490	108.8549
$ZC_6[5,9]$	155.5456	282.0612	148.2364	859.7601	122.3128
$ZC_6[5,10]$	168.6165	308.3171	161.5894	935.8338	131.6698
$ZC_6[5,11]$	187.1002	345.4395	180.2012	1039.4182	145.0638
$ZC_6[5,12]$	200.1333	372.0528	193.6316	1115.2485	154.3475
$ZC_6[5,13]$	218.6333	409.2243	212.2461	1218.8922	167.7026
$ZC_6[5,14]$	231.6943	436.5013	225.9625	1294.9403	176.9359
$ZC_6[5,15]$	250.1350	474.6518	244.8417	1398.2733	190.2373

We proceed by utilizing the cftool to perform curve fitting on the data presented in Table 3. Following this step, our MatLab code outputs the results that follows, alongwith 95% coefficients’ confidence intervals:

$$\begin{aligned} En_{H}(ZC_6[5,n])= & 4159 _{\pm 240900}\sin(0.003804_{\pm 0.2206}n + 0.002858{\pm 0.16425}),\\ Es_{GA^2}(ZC_6[5,n]))= & 0.2787_{\pm 0.2318}n^2 +25.61_{\pm 4.03}n + 26.65_{\pm 15.15},\\ En_{GA^2}(ZC_6[5,n])= & 0.08838_{\pm 0.11369}n^2 +14.05 _{\pm 1.98}n +13.48_{\pm 7.428},\\ En_{DD}(ZC_6[5,n])= & 2.9\times 10^4_{\pm 2.3\times 10^6}\sin(0.0031_{\pm 0.2}n-0.001_{\pm 0.118}),\\ Es_{ABC^2}(ZC_6[5,n])= & -0.03783_{\pm 0.09} n^2 + 12.2_{\pm 1.7} n + 14.72_{\pm 6.5}.\\ \end{aligned}$$

These findings will significantly enhance the accuracy of correlating the the two thermodynamic properties of $ZC_6[5,n]$.

## Applicability to carbon nanostructures: one-hexagonal nanocones

Within carbon nanostructures lies a captivating phenomenon: the presence of hollow carbon configurations. Among these, carbon nanocones stand out, resembling nano-caps situated at the ends of nanotubes, alongside nanotubes and fullerenes. They can also be seen as independent structures atop a flattened graphite surface. The formation of a carbon nanocone typically involves extracting a wedge from a graphite sheet, rolling the remaining segment around its peak, and subsequently linking the two exposed sides. For a study in electronic structure of these pentagonal nanocones, see^72^.

When the apex angle surpasses 60°, nanocones develop a hexagonal shape at their peak. These nanocones, featuring a singular hexagon at their apex, are categorized as carbon nanocones and are referred to as having a central core.

**Fig. 5:**
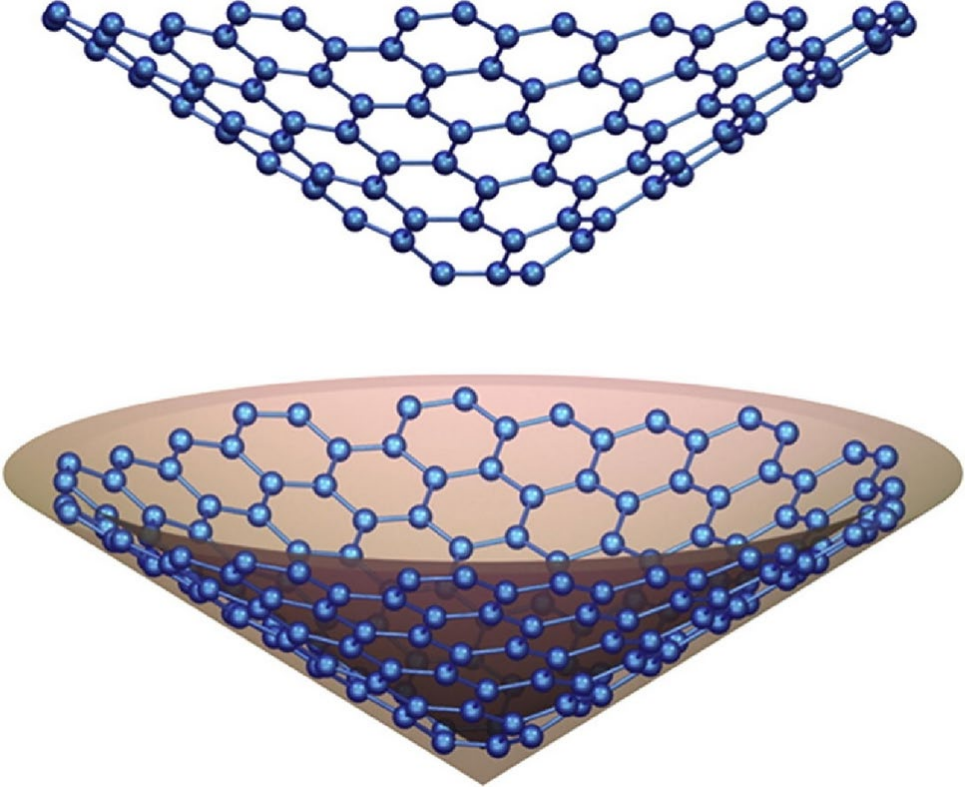
Visual representations depicting a nanocone with a single hexagonal base from different angles in three dimensions.

The illustration depicted in Fig. 5 showcases two distinct 3D perspectives of a nanocone with a singular hexagonal base. For a computational study on spectral descriptors of one-pentagonal nancones, we refer to^73^.

**Fig. 6:**
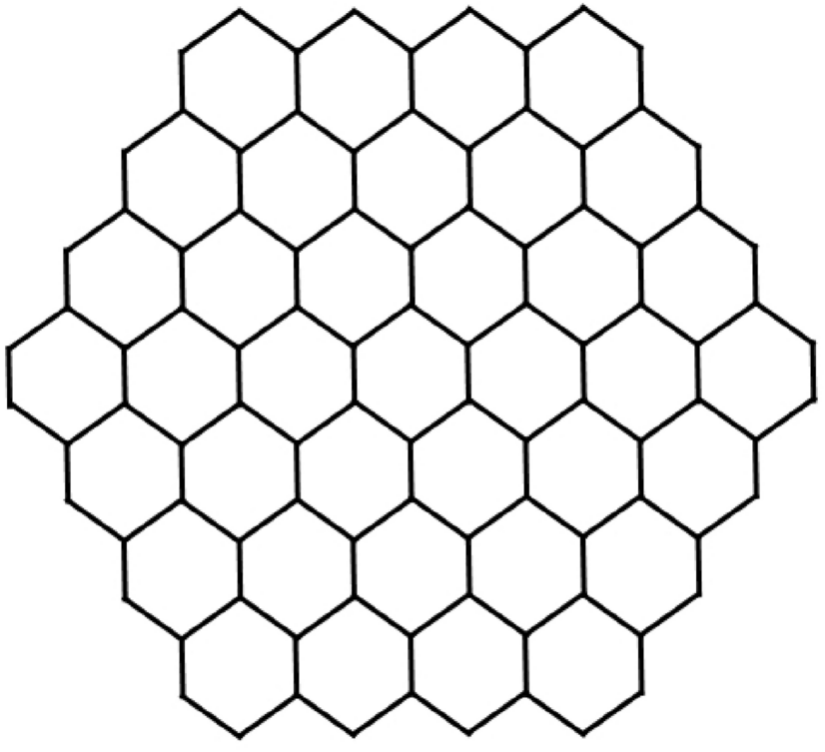
The 3-dimensional $CNC_6[3]$ nanocone.

We represent a nanocone with a conical surface covered by *n* layers of hexagons as $CNC_6[n]$. Here, *n* signifies the number of hexagonal layers, while the subscript “6” indicates that the core of the nanocone forms a hexagon. Figure 6 illustrates the $CNC_6[3]$ nanocone in three dimensions.

In Sect. A computer-dependent computational technique, we utilize our computational approach to determine the top five spectrum-based topological descriptors as illustrated in Table 5 for a single-hexagonal nanocone denoted as $CNC_6[n]$. The results of these computations for $CNC_6[n]$ are presented in Table 4 for values of *n* ranging from 1 to 10.

**Table 4:** The best 5 distance-spectral graphical invariant for $CNC_6[n]$ with $1\le n\le 10$.

$CNC_6[n]$	$En_H$	$Es_{GA^2}$	$En_{GA^2}$	$En_{DD}$	$Es_{ABC^2}$
$CNC_6{[}1{]}$	32.6030	63.6081	33.5744	166.0818	28.3634
$CNC_6{[}2{]}$	75.8024	151.8531	77.6014	410.3461	58.4956
$CNC_6{[}3{]}$	136.6019	280.5523	141.0622	760.2151	100.2979
$CNC_6{[}4{]}$	214.9346	442.9552	221.5188	1215.3431	154.4797
$CNC_6{[}5{]}$	310.9049	644.8226	321.0271	1776.4108	220.5881
$CNC_6{[}6{]}$	424.5764	879.8015	436.9177	2443.6358	298.7547
$CNC_6{[}7{]}$	556.0342	1150.0168	571.3838	3217.4930	388.8993
$CNC_6{[}8{]}$	705.2066	1465.6511	726.9697	4097.6440	490.8686
$CNC_6{[}9{]}$	870.5932	1801.4729	893.6640	5077.6889	604.0102
$CNC_6{[}10{]}$	1055.0350	2190.3040	1085.3135	6170.4290	730.0064

We utilized the MatLab R2013a version along with cftool to analyze the data provided in Table 4. Our MatLab script produced the subsequent outcomes, accompanied by 95% confidence intervals for the coefficients:

$$\begin{aligned} En_{H}(CNC_6[n])= & 4.91 \times 10^4_{\pm 3.2 \times 10^8} \sin ({0.002311_{\pm 15.2} n - 0.003792_{\pm 24.9}}), \\ Es_{GA^2}(CNC_6[n]))= & 236.2_{\pm 75.1}n - 391.9_{\pm 466.1},\\ En_{GA^2}(CNC_6[n])= & 116.8_{\pm 37.17}n - 191.5_{\pm 230.5},\\ En_{DD}(CNC_6[n])= & 3914_{\pm 4598} \sin ({0.1822_{\pm 0.5}n - 0.2928_{\pm 1.3}}), \\ Es_{ABC^2}(CNC_6[n])= & 77.9_{\pm 24.6} n - 121.4_{\pm 152.9}. \end{aligned}$$

These findings will aid in correlating the two thermodynamic characteristics of the one-hexagonal nanocones with much greater precision.

## Discussion and concluding remarks

This study delves into the correlation capabilities of widely employed distance-related spectral descriptors, which rely on eigenvalues of matrices corresponding to distances in molecular structures. Within this study, we put forward 3 novel matrices, and our findings indicate that distance-spectral invariants generate by those novel matrices surpass existing, extensively researched invariants. Initially, we present a unified computing methodology for calculating these prevalent distance-related spectral descriptors. This approach is then applied to compute commonly utilized distance-related spectral descriptors, examining their estimation ability with experimental values of *Ent* and $H_c$ (the chosen thermodynamic characteristics).

We evaluated the effectiveness of a graphical invariant, as outlined in Table 1, by analyzing the average $\rho _{\textrm{mean}}$ of two correlation values. Our straightforward criteria is that the nearer the $\rho _{\textrm{mean}}$ is to one, the more effective the graphical index performs. Using this metric, we have identified and ranked the top five graphical indices with the highest performance. The 4 best distance-spectral graphical invariants are presented in Table 5. Additionally, Table 6 offers a comprehensive statistical analysis for these proffered distance-spectral invariants.

**Table 5:** The 5 best distance-spectral graphical invariants.

Graphical invariant	Placement
$En_H$, Eq. (11)	1
$Es_{GA^2}$, Eq. (31)	2
$En_{GA^2}$, Eq. (30)	3
$En_{DD}$, Eq. (20)	4
$Es_{ABC^2}$, Eq. (28)	5

**Table 6:** Structure-property regression models and other statistical parameters of the 5 best distance-spectral graphical invariants.

S. no	Index	Regression model	Statistics
1	$En_H$	$0.2039_{\pm {0.021}}H_c -0.0821_{\pm {0.019}}Ent + 13.1214_{\pm 3.850}$	$r^2=0.9926,~\rho =0.9963,~s=0.6080$
2	$Es_{GA^2}$	$0.4319_{\pm {0.06}}H_c -0.2083_{\pm {0.05}}Ent + 36.2320_{\pm {10.54}}$	$r^2=0.9833,~\rho = 0.9916,~s= 1.6643$
3	$En_{GA^2}$	$0.2151_{\pm {0.029}}H_c - 0.0977_{\pm {0.026}}Ent + 17.4063_{\pm {5.276}}$	$r^2=0.9849,~\rho =0.9924,~s=0.8333$
4	$En_{DD}$	$1.2683_{\pm {0.15}}H_c - 0.6159_{\pm {0.14}}Ent + 94.3422_{\pm {+-28.24}}$	$r^2=0.9859,~\rho =0.9929,~s= 4.4598$
5	$Es_{ABC^2}$	$0.1330_{\pm {0.021}}H_c - 0.0389_{\pm {0.019}}Ent + 9.1318_{\pm {3.799}}$	$r^2=0.9882,~\rho =0.9941,~s= 0.6$

Our findings strongly support the efficacy of our recently developed distance-related spectral descriptors. Among them, the 2${\text{nd}}$ ABC Estrada inariant demonstrates superior performance. Additionally, two other novel spectral descriptors, derived from the 2${\text{nd}}$ geometric-arithmetic (GA), have risen to prominence, ranking within the top five. These descriptors include the $Es_{GA^2}$ as well as $En_{GA^2}$ of graphs. Moreover, established eigenvalues-dependent invariants from existing literature, such as the degree-distance and Harary energies, have also earned positions among the top five distance-related spectral descriptors. These outcomes underscore the importance of incorporating the $En_{ABC^2}$ and $En_{H}$ into structure-property modeling, warranting further exploration of their utility.

The information presented in Table 1 reveals a mix of positive and negative findings. Regarding positive outcomes, certain lesser-known distance-related spectral descriptors, such as the Harary energy and the degree-distance energy, exhibited exceptional performance despite their limited recognition among researchers. Conversely, some widely accepted and esteemed distance-related spectral descriptors, such as the distance energy, Estrada index, distance Laplacian, signless Laplacian energies, and Estrada indices, did not meet the expectations within the community of chemical graph theorists and theoretical chemists.

In practical terms, graphical indices with correlation coefficients below 0.95 are unsuitable for structure-property modelling. Notably, widely recognized distance-spectral invariants like $En_{\mathscr {D}}$, $Es_{\mathscr {D}}$, $En_{\mathscr {L}}$, $En_{\mathscr {Q}}$ and respective Estrada invariants, $En_{Sz}$, $En_{\mathscr {S}}$ and respective Estrada invariants, etc., exhibit correlated values $<0.95$, indicating significant inadequacy for application purposes. Therefore, endorsing these descriptors for use in structure-property modelling would be ill-advised.

When it comes to models depicting the relationship between structure and properties, we favor the recently introduced $Es_{GA^2}$ and the $En_{H}$. Both of these exhibit correlated values exceeding 0.95. Despite any doubts about their reliability, the findings endorse ongoing utilization of these indices in structure-property modelling.

**Fig. 7:**
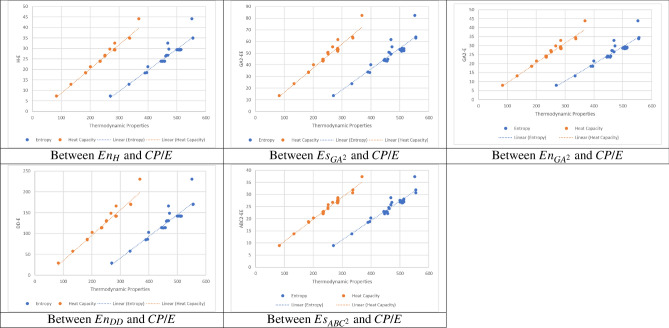
Scatter plots illustrating the relationship between the top five spectral descriptors related to distance and the heat capacity/entropy of 30 lower PHs.

Following this, we showcase scatter plots illustrating the top five spectral descriptors associated with distance in relation to heat capacity and entropy for a chosen subset of 30 lower polycyclic hydrocarbons. Figure 7 exhibits these visual representations alongside their respective linear regression models, elucidating the dispersion of data and the disparities between actual and correlated data points.

In conclusion, when incorporating them into structure-property relationship models, we exclusively favor utilizing the second atom-bond connectivity energy and the Harary energy, given that both exhibit correlation coefficients exceeding 0.95. Despite any existing skepticism, the findings strongly support the ongoing inclusion of the second atom-bond connectivity index in both structure-activity and structure-property models.

## Data Availability

The datasets used and/or analysed during the current study available from the corresponding author on reasonable request
